# Screening for cognitive impairment in late onset depression in a Brazilian sample using the BBRC-Edu

**DOI:** 10.1590/S1980-57642012DN06020004

**Published:** 2012

**Authors:** Tânia Maria da Silva Novaretti, Marcia Radanovic, Ricardo Nitrini

**Affiliations:** 1 State University Paulista Julio de Mesquita Filho, Faculty of Sciences, Marília Campus, Department of Speech, Marília SP, Brazil.; 2 Behavioral and Cognitive Neurology Unit, Department of Neurology, University Department of Neurology, University of São Paulo School of Medicine, São Paulo SP, Brazil.; 3 Behavioral and Cognitive Neurology Unit, Department of Neurology, and Cognitive Disorders Reference Center (CEREDIC), Hospital das Clínicas of the University of São Paulo School of Medicine, São Paulo SP, Brazil.

**Keywords:** Alzheimer's disease, CERAD, BBRC-Edu, depression, screening test, cognition, elderly

## Abstract

Depression and dementia are the most prevalent neuropsychiatric disorders in the elderly population. Alzheimer's disease is the leading cause of dementia in most countries, being responsible for more than half of all dementia cases. Late-onset depression is a frequent cause of cognitive decline in the elderly. Differentiating between cognitive impairment secondary to depression and incipient dementia poses a challenge in the clinical setting.

**Objective:**

To evaluate the performance of elderly depressed patients using the BBRC-Edu.

**Methods:**

We studied 25 patients with late onset depression (mean age: 73.6 y (6.6); schooling: 9.1 y (5.7)) and 30 patients with mild AD (mean age 76.6 y (5.4); schooling: 7.5 y (7.1)), who were compared to a control group of 30 healthy elderly (mean age 73.8 y (5.8); schooling: 9.1 y (5.4)) using the CERAD and BBRC-Edu batteries.

**Results:**

For the CERAD battery, depressed patients performed better than AD patients on all tasks (p<0.0001) except for Constructional Praxis (p>0.05), and performed poorer than controls on verbal fluency (animals) and Word List Recall tasks (p<0.0001). For the BBRC-Edu, depressed patients performed better than AD patients on all tasks (p<0.0001) except for Digit Span (direct order) (p=0.076) and Incidental Memory (p>0.05), and performed worse than controls on Learning (second presentation) and verbal fluency (fruits) tasks (p<0.0001).

**Conclusion:**

Overall performance on the BBRC-Edu allowed differentiation of controls and depressed patients from AD patients.

## Abstract (pt)

Depressão e demência são os transtornos neuropsiquiátricos de maior prevalência na população idosa. A doença de Alzheimer é a principal causa de demência na maioria os países, sendo a responsável por mais da metade dos casos de demência. Depressão de início tardio é uma causa frequente de declínio cognitivo no idoso. A diferenciação entre transtorno cognitivo secundário à depressão e demência em fase inicial é um desafio na prática clínica.

**Objetivo:**

Avaliar o desempenho de pacientes idosos deprimidos usando a Bateria Breve de Rastreio Cognitivo (BBRC-Edu).

**Métodos:**

Estudamos 25 pacientes com depressão de início tardio, (idade média 73,6 (6,6); escolaridade: 9,1 (5,7)) e 30 pacientes com DA leve (CDR=1), (idade média 76,6 (5,4); escolaridade: 7,5 (7,1)). O desempenho de ambos os grupos foi comparado a um grupo controle de 30 idosos sadios (idade média 73,8 (5,8); escolaridade: 9,1 (5,4)).

**Resultados:**

Na Bateria CERAD pacientes deprimidos tiveram desempenho melhor que pacientes com DA em todos os testes (p<0,0001) exceto para Praxia Construcional (p>0,05), e desempenho pior que os controles na fluência verbal (animais) e Recordação da Lista de Palavras (p<0,0001). Na BBRC-Edu, pacientes deprimidos tiveram melhor desempenho comparados aos pacientes com DA em todos os testes (p<0,0001) exceto para Spam de Dígitos (ordem direta) (p=0,076) e Memória Incidental (p>0,05), e tiveram desempenho pior que os controles na Aprendizagem (segunda apresentação) e na fluência verbal (frutas) (p<0,0001).

**Conclusão:**

A Bateria Breve de Rastreio Cognitivo permite a diferenciação de controles e pacientes com transtorno depressivo, de pacientes com doença de Alzheimer.

## INTRODUCTION

Depression and dementia are two of the most prevalent neuropsychiatric diseases in the elderly, yet this population is far from homogeneous in terms of cognitive decline and depressive symptoms. Major depressive disorder is common in the general population having an estimated lifetime prevalence of 15-17%.^1^ Clinically significant depressive symptoms are present in 11-39% of older adults^2-4^ while cognitive impairment is estimated to affect 17-36% of adults aged over 65 years.^4-6^ Elderly demented patients have shown an even higher prevalence of depression (24%) in epidemiological studies.^7^

Age-related cognitive decline is usually characterized by mild impairment in a number of cognitive domains,^8^ typically manifested as changes in episodic memory or reduced ability to access information stored in long-term memory^9,10^ resulting from decreased speed of the central processing required to encode and retrieve information.^11^ In this population, working memory becomes affected^12,13^ while retention usually remains preserved^8,12^ as does implicit memory.^14^ Other changes associated with normal aging include mild deficits in language functioning (naming, verbal fluency), visuospatial abilities, perceptual speed and executive functioning.^15-17^ Amongst patients who suffer from late-life depression, 20-50% display deficits across a wide range of cognitive domains including processing speed,^18^ attention and executive functions^19,20^ as well as episodic and working memory.^21^

Furthermore, the level of generalized cognitive impairment associated with depression can be so marked in the elderly that depression is often misdiagnosed as dementia. Indeed, 9-43% of elderly persons who have depression subsequently develop some kind of dementia.^22^ This cognitive impairment can persist despite improvement in depressive symptoms.^2,23^ However, the true pattern of cognitive impairment in late-onset depression remains a matter of controversy because most studies available are modest or small in size, and cognitive deficits tend to differ in both nature and severity, between and within studies.^24^

In view of the above-mentioned interrelationship between depression and AD, diagnosing dementia in depressed patients, regardless of depression, poses a challenge.^25^ The aim of this study was to evaluate the performance of elderly depressed patients, healthy elderly, and patients with early stage AD using the BBRC-Edu, a screening battery fully developed by Brazilian researchers.^26^ To the best of our knowledge, this is the first study to employ the BBRC-Edu in the assessment of depressed elderly.

## METHODS

Thirty patients with mild AD, 25 with depression and 30 control subjects were studied. All participants were native speakers of Brazilian Portuguese, aged over 60 years, and had at least 2 years of formal education. Subjects with a past history or neurological evidence of stroke, neurodegenerative disorders, head injury, serious non-compensated medical illness, drug abuse, hearing, visual or motor impairment that could have affected their cognitive performance were not included. The control group was defined as subjects living in the community, who fulfilled the MOANS criteria^27^ and achieved normal scores on the cognitive evaluation (corrected for age and schooling). The diagnosis of depression was determined according to the DSM-IV criteria,^28^ while the diagnosis of Probable AD was based on the NINCDS-ADRA criteria.^29^ Only mild AD patients (CDR 1) were included.^30^

The cognitive evaluation entailed application of the Brazilian version of the Consortium to Establish a Registry for Alzheimer's Disease Battery (CERAD).^31,32^ Depressive symptoms were evaluated using the Geriatric Depression Scale (GDS),^33^ the Hamilton Depression Scale (HAM-D),^34^ and the Montgomery & Asberg Depression Rating Scale (MADRS).^35,36^ Functional abilities were assessed using the Pfeffer Functional Activities Questionnaire (PFAQ).^37^ Depressed patients were evaluated within one week of introducing antidepressant medication.

After the initial evaluation, subjects were classified into controls, depression or mild AD. Participants were then assessed using the Brief Cognitive Battery-Edu (BBRC-Edu), which was developed in Brazil and has proven suitable for screening cognitive impairment in the Brazilian population, being less impacted by educational level.^26^

The performance of the three groups on each task was compared using one-way ANOVA with Tukey's post-hoc test. All statistical analyses were performed using the SPSS for Windows® 10.0.1 software, and a significance level of 0.05 was adopted.

This study was approved by the local Research Ethics Committee (Hospital das Clínicas – University of São Paulo School of Medicine) and all participants or their legal representatives signed an informed consent prior to enrolling on the study.

## RESULTS

The demographic and clinical characteristics of the sample are shown in Table 1. The mean values for age and schooling level were similar across the three groups. Women predominated in the depression and AD groups. AD patients had lower scores on the PFAQ, as expected. Controls and Depression patients did not differ for PFAQ scores. All groups differed in GDS, HAM-D and MADRS scores, with patients from the Depression group exhibiting the highest scores while patients from the AD group had scores between those of the Control and Depression groups.

**Table 1:** Demographic and clinical characteristics of the sample.

Variable		CG Mean (SD) range	DG Mean (SD) range	AD Mean (SD) range	p (two-tailed) Multiple comparison
Age		73.8 (5.8) 60-87	73.6 (6.6) 65-93	77.6 (5.4) 60-85	0.055*
Schooling		9.1 (5.4) 2-23	9.1 (5.7) 2-20	7.5 (7.1) 2-33	0.519*
Gender	M	16	20	23	0.0005**
	F	14	5	7	CG × DG p=0.005
					CG × AD p=0.006
PFAQ		0 (0)0-0	1.2 (2)0-5	19.9 (5.2)5-28	<0.0001* CG & DG × AD (p < 0.0001)
GDS		2.2 (2.6)0-9	24.6 (4.6)16-30	10.3 (4.5)3-17	<0.0001* all groups differ
HAM-D		2.4 (3.9)0-20	32.8 (5.4)27-46	14.6 (8.3)1-26	<0.0001* all groups differ
MADRS		2.4 (5)0-24	36.3 (7.2)30-51	16.5 (8.4)2-28	<0.0001* all groups differ

CG: control group; DG: depression group; F: female; M: male; NA: not applicable;

* ANOVA with Tukey's post hoc test;

** Chi-square test.

Table 2 shows performance of the three groups on the CERAD battery. Depressed patients performed better than AD patients on all tasks except for Constructional Praxis, but performed poorer than controls on verbal fluency (animals) and Word List Recall tasks.

**Table 2:** Performance of the three groups on the CERAD battery.

Variable	CG Mean (SD) range	DG Mean (SD) range	AD Mean (SD) range	p (two-tailed)* Multiple comparison
Verbal fluency (Animals)	18.1 (4)14-30	15.1(2.3)12-20	9.7 (2.8)0-14	<0.0001 all differ
Naming 15 items	13.2 (1.7)10-15	12.9 (1.7)10-15	10.6 (2.1)5-14	<0.0001 CG & DG × AD (p<0.0001)
MMSE	27.2 (1.4)23-30	26 (2.3)21-30	20.7 (3.2)13-26	<0.0001 CG & DG × AD (p<0.0001)
Word List Memory	15.8 (3)12-24	15.3 (3.1)18-24	7.9 (3.8)2-16	<0.0001 CG & DG × AD (p<0.0001)
Constructional Praxis	9.7 (1.7)7-11	9.2 (1.9)7-13	8.1 (2.9)4-13	0.022 CG × AD (p<0.018)
Word List Recall	4.7 (1.6)3-10	3.4 (1.2)1-7	0.7 (0.9)0-3	<0.0001 all differ
Word List Recognition	8.9 (0.8)7-10	8.3 (1.5)5-10	4.3 (2.8)0-10	<0.0001 CG & DG × AD (p<0.0001)
Praxis Recall	7.6 (3.1)2-11	6.3 (2.9)2-11	1.5 (2.4)0-10	<0.0001 CG & DG × AD (p<0.0001)

CG: control group; DG: depression group; F: female; M: male; NA: not applicable;

* ANOVA with Tukey's post hoc test.

On the BBRC-Edu, depressed patients performed better compared with AD patients on all tasks except for Digit Span (direct order) and Incidental Memory, yet performed worse than controls on Learning (second presentation), and verbal fluency (fruits) tasks. For the two latter tasks, depressed patients showed an intermediate performance compared to controls and AD patients (Table 3).

**Table 3:** Performance of the three groups on the BBRC-Edu battery.

Variable		CG Mean (SD) range	DG Mean (SD) range	AD Mean (SD) range	p (two-tailed)* Multiple comparison
**Digit Span**	Direct order	8.8 (1.7)6-12	8.9 (1.4)6-12	7.9 (1.9)5-11	0.076
	Reverse order	4.5 (1.5)2-8	4.1 (1.5)2-8	2.8 (1.6)1-8	0.0001 CG × AD (p<0.0001) DG × AD (p=0.004)
**Learning and memory**	Perception/Naming	10 (0)10-10	10 (0)10-10	9.8 (0.3)9-10	0.020 CG & DG × AD (p=0.037)
	Incidental memory	4.7 (1.4)3-9	4.1 (1.5)2-7	3.5 (1.7)0-7	0.011 CG × AD (p=0.008)
	Learning 1	7 (1.4)5-10	6.7 (2)3-10	4.6 (1.7)1-8	<0.0001 CG & DG × AD (p<0.0001)
	Learning 2	8.9 (0.9)7-10	7.4 (1.9)3-10	5.2 (1.3)2-8	<0.0001 all differ
	Delayed recall	7.5 (1.7)4-10	6.3 (2.3)2-10	2.4 (2.2)0-7	<0.0001 CG × AD (p<0.001) DG × AD (p<0.0001)
	Picture recognition	9.7 (0.5)8-10	9.5 (0.8)7-10	6.9 (2.8)0-10	<0.0001 CG & DG × AD (p<0.0001)
	Verbal fluency (Fruits)	15.6 (2.4)13-23	13 (2)7-16	9 (2.7)4-16	<0.0001 all differ
	Clock drawing	9.3 (1.6)2-10	8.4 (2)3-10	4.9 (2.5)2-10	<0.0001 CG & DG × AD (p<0.0001)

CG: control group; DG: depression group; F: female; M: male; NA: not applicable;

* ANOVA with Tukey's post hoc test.

## DISCUSSION

The choice of psychometric tests for use in Portuguese-speaking people classified into well-defined groups is limited.^32^ It is difficult to discriminate early dementia from depression based on clinical and neuropsychological evidence^37^ because similar cognitive and affective problems can occur in both mild AD and "depressive pseudo-dementia".^39^ The hallmark of AD is marked impairment in episodic memory, which may exist years before a clinical diagnosis of dementia is established.^40,41^ However, semantic impairments also occur in approximately 50% of patients with mild AD where semantic memory may be the most important cognitive domain for performing everyday skills.^10,42^

Depression is particularly associated with deficits in executive control processes.^43,44^ Patients with late-onset depression have demonstrated impairments in executive functioning, processing speed, as well as episodic and semantic memory.^45^ Notwithstanding, few studies in the literature on late-onset depression have investigated semantic memory. However, semantic memory tasks often require preserved executive function. Category fluency for example, although generally regarded as a valid measure of semantic memory, relies heavily on initiation, which may be impaired in depression.^45^ In addition, the individual under test is required to perform this task within a set time limit, which makes processing speed an important factor for good performance.^46^ In the present study, depressed patients consistently displayed impairment in verbal fluency, both for animal and fruits categories.

The presence of depression-related deficits in geriatric depression has been noted in episodic memory.^23,47-49^ Sheline et al. 200649 and Delaloyle et al. 2008^50^ have suggested that while slowed processing speed appears to be the core cognitive deficit in late-onset depression, it was closely followed by executive dysfunction. However, these authors hold that episodic memory impairment cannot be explained solely by processing resource decreases in late-onset depression patients. Recent data suggest that the reduction in processing resources is a primary cognitive decrement in late-onset depression, which subsequently induces higher-level cognitive deficits.^18-21,49^ For instance, besides memory, impaired language abilities in old age have been associated with a reduction in processing resources, such as processing speed, working memory span and inhibition capacity. Processing speed, which denotes the pace at which elementary cognitive operations are carried out, is reduced in late-onset depression.^21^ Depression-related variance on higher-order cognitive tests is also thought to be mediated by reduced working memory span, which is able to maintain or suppress the activation of long-term memory units and allocation of intentional resources. Finally, inefficient inhibitory processes permit the entry and maintenance of irrelevant information in the working memory, affecting subsequent cognitive performance.^50^ In this study, depressed patients did not differ from controls in cued recall memory tasks (recognition) for verbal and non-verbal stimuli, thus suggesting an impairment of self-generated retrieval strategies, which may be secondary to lower processing speed associated to executive dysfunction.

In the clinical setting, it may be difficult to differentiate depression from incipient AD in patients with cognitive impairment. Brief cognitive tests may be helpful in assessing patients with suspected dementia in primary care facilities provided these instruments are sufficiently accurate. By comparing the performance of controls, depressed patients and mild AD patients using a well-accepted battery (CERAD) plus a battery developed in Brazil (BBRC-Edu), we found that the latter was suitable for detecting mild cognitive impairment in depressed patients and also proved able to discriminate this group from AD patients. Further studies are warranted in order to establish the degree of agreement between the BBRC-Edu and other broadly used screening tests, such as the MMSE and CERAD.

## References

[1] APA (1994). Diagnostic and Statistical Manual of Mental Disorders.

[2] Steffens DC, Skoog I, Norton MC (2000). Prevalence of depression and its treatment in an elderly
population: The Cache County study. Arch Gen Psychiatry.

[3] Copeland JR, Beekman AT, Braam AW (2004). Depression among older people in Europe: The EURODEP
Studies. World Psychiatry.

[4] Lee Y, Shinkai S (2005). Correlates of cognitive impairment and depressive symptoms among
older adults in Korea and Japan. Int J Geriatr Psychiatry.

[5] Rait G, Fletcher A, Smeeth L (2005). Prevalence of cognitive impairment: results from the MCR trial of
assessment and management of older people in the community. Age Aging.

[6] Graciani A, Banegas JR, Guallar-Castillon P, Domínguez-Rojas V, Rodríguez-Artalejo F (2006). Cognitive assessment of the non-demented elderly community
dwellers in Spain. Dement Geriatr Cogn Disord.

[7] Lyketsos CG, Steinberg M, Tschanz JT, Norton MC, Steffens DC, Breitner JC (2000). Mental and behavioral disturbances in dementia: findings from the
Cache Country Study on Memory in Aging. Am J Psychiatry.

[8] Petersen RC, Stevens JC, Ganguli M, Tangalos EG, Cummings JL, DeKosky ST (2001). Practice parameter: Early detection of dementia: Mild cognitive
impairment (an evidence-based review). Neurology.

[9] Dixon RA, Wahlin A, Maitland SB, Hultsch DF, Hertzog C, Backman L (2004). Episodic memory change in late adulthood: generalizability across
samples and performances indices. Memory Cognit.

[10] Amieva H, Goff ML, Millet X (2008). Prodromal Alzheimer's disease: successive emergence of the
clinical symptoms. Ann Neurol.

[11] Carlson MC, Xue QL, Zhou J, Fried LP (2009). Executive decline and dysfunction precedes declines in memory:
the women's health and aging study II. J Gerontol.

[12] Rabinowitz JC, Craik FI (1986). Prior retrieval effects in young and old adults. J Gerontol.

[13] Hultsch DF, Hertzog C, Small BJ, McDonald-Miszczak L, Dixon RA (1992). Short-term longitudinal change in cognitive performance in later
life. Psychol Aging.

[14] Ritchie K, Touchon J, Ledesert B, Leibovici D, Gorce AM (1997). Establishing the limits and characteristics of normal age-related
cognitive decline. Rev Epidemiol Sante Publique.

[15] Rubin EH, Storandt M, Miller JP (1998). A prospective study of cognitive function and onset of dementia
in cognitively healthy elders. Arch Neurol.

[16] Salthouse T (1989). Mechanisms of age-cognition relations in adulthood.

[17] Schaie KW (1989). Perceptual speed in adulthood: Cross-sectional and longitudinal
studies. Psychol Aging.

[18] Butters MA, Whyte EM, Nebes RD (2004). The nature and determinants of neuropsychological functioning in
late-life depression. Arch Gen Psychiatry.

[19] Lockwood KA, Alexopoulos GS, Van Gorp WG (2002). Executive dysfunction in geriatric depression. Am J Psychiatry.

[20] Rapp MA, Dahlman K, Sano M, Grossman HT, Haroutunian V, Gorman JM (2005). Neuropsychological differences between late-onset and recurrent
geriatric major depression. Am J Psychiatry.

[21] Nebes RD, Butters MA, Mulsant BH (2000). Decreased working memory and processing speed mediate cognitive
impairment in geriatric depression. Psychol Med.

[22] Stek ML, Van Exel E, Van Tilburg W, Westendorp RGJ, Beekman ATF (2002). The prognosis of depression in old age: outcome six to eight
years after clinical treatment. Aging Ment Health.

[23] Bhalla RK, Butters MA, Mulsant BH (2006). Persistence of neuropsychological deficits in the remitted state
of late-life depression. Am J Geriatr Psychiatry.

[24] Rose EJ, Ebmeier KP (2006). Pattern of impaired working memory during major
depression. J Affect Disord.

[25] Novaretti TMS, D'Avila Freitas MI, Mansur LL, Nitrini R, Radanovic M (2011). Comparison of language impairment in late-onset depression and
Alzheimer's disease. Acta Neuropsychiatr.

[26] Nitrini R, Caramelli P, Porto CS, Fichman HC, Formigoni AP (2007). Brief cognitive battery in the diagnosis of mild Alzheimer's
disease in subjects with medium and high levels of education. Dement Neuropsychol.

[27] Smith GE, Ivnik RJ, Petersen RC (2003). Normative neuropsychology. Mild Cognitive Impairment.

[28] Spitzer RL, Williams JB, Gibbon M, First MB (1992). The Structured Clinical Interview for DSM-III-R (SCID). I:
History, rationale and description. Arch Gen Psychiatry.

[29] McKhann G, Drachman D, Folstein M, Katzman R, Price D, Stadian EM (1984). Clinical diagnosis of Alzheimer's disease: Report of the
NINCDS-ADRDA Work Group under the auspices of Department of Health and Human
Services Task Force on Alzheimer's disease. Neurology.

[30] Hodges JR, Salmon DP, Butters N (1992). Semantic memory impairment in Alzheimer's disease: failure of
access or degraded knowledge. Neuropsychologia.

[31] Morris JC, Heyman A, Mohs RC (1989). The Consortium to Establish a Registry for Alzheimer's Disease
(CERAD). Part I: Clinical and neuropsychological assessment for Alzheimer's
disease. Neurology.

[32] Bertoluci PHF, Okamoto IH, Brucki SMD, Siviero MO, Toniolo Neto J, Ramos LR (2001). Applicability of the CERAD neuropsychological battery to
Brazilian elderly. Arq Neuropsychiatr.

[33] Yesavage JA, Brink TL, Rose TL (1983). Development and validation of geriatric depression screening
scale: a preliminary report. J Psychiatr Res.

[34] Hamilton M (1960). A rating scale for depression. J Neurol Neurosurg Psychiatry.

[35] Montgomery AS, Asberg M (1979). New depression scale designed to be sensitive to
change. Br J Psychiatry.

[36] Moreno RA, Moreno DH (1998). Escalas de depressão de Montgomery & Åsberg
(MADRS) e de Hamilton (HAM-D). Rev Psiq Clin.

[37] Pfeffer RI, Kurosaki TT, Harrah CH Jr, Chance JM, Filos S (1982). Measurement of functional activities in older adults in the
community. J Gerontol.

[38] Foldi NS, Brickman AM, Schaefer LA, Knutelska ME (2003). Distinct serial position profiles and neuropsychological measures
differentiate late life depression from normal aging and Alzheimer's
disease. Psychiatry Res.

[39] Swainson R, Hodges JR, Galton CJ (2001). Early detection and differential diagnosis of Alzheimer's disease
and depression with neuropsychological tasks. Dement Geriatr Cogn Disord.

[40] Backman L, Small B, Fratiglioni L (2001). Stability of the preclinical episodic memory deficit in
Alzheimer's disease. Brain.

[41] Chen P, Ratcliff G, Belle SH (2001). Patterns of cognitive decline in presymptomatic Alzheimer
disease: a prospective community study. Arch Gen Psychiatry.

[42] Perry RJ, Hodges JR (2000). Relationship between functional and neuropsychological
performance in early Alzheimer's disease. Alzheimer Dis Assoc Disord.

[43] Henry JD, Crawford JR (2005). Verbal fluency performance in dementia of the Alzheimer type; a
meta-analysis. Neuropsychologia.

[44] Henry JD, Crawford JR (2005). A meta-analytic review of verbal fluency deficits in
depression. J Clin Exp Neuropsychol.

[45] Herrmann LL, Goodwin GM, Ebmeier KP (2007). The cognitive neuropsychology of depression in the
elderly. Psychol Med.

[46] Norris MP, Blankenship-Reuter l, Snow-Turek AI, Finch J (1995). Influence of depression on verbal fluency
performance. Age Cog.

[47] Portela MJ, Marcos T, Rami L, Navarro V, Gasto C, Salamero M (2003). Residual cognitive impairment in late-life depression after a
12-month period follow-up. Int J Geriatr Psychiatry.

[48] King DA, Cox C, Lyness JM, Conwell Y, Caine ED (2006). Quantitative and qualitative differences in the verbal learning
performance of elderly depressives and healthy controls. J Int Neuropsychol Soc.

[49] Sheline YI, Barch DM, Garcia K (2006). Cognitive function in late life depression: relationships to
depression severity, cerebrovascular risk factors and processing
speed. Biol Psychiatry.

[50] Delaloye C, Baudois S, Bilbao F (2008). Cognitive impairment in late-onset depression. Limited to a
decrement in information processing resources?. Eur Neurol.

